# Type 2 diabetes and glycemic traits are not causal factors of delirium: A two-sample mendelian randomization analysis

**DOI:** 10.3389/fgene.2023.1087878

**Published:** 2023-02-21

**Authors:** Jing Li, Mingyi Yang, Pan Luo, Gang Wang, Buhuai Dong, Peng Xu

**Affiliations:** ^1^ Department of Anesthesiology, Honghui Hospital, Xi’an Jiaotong University, Xi’an, Shaanxi, China; ^2^ Department of Joint Surgery, Honghui Hospital, Xi’an Jiaotong University, Xi’an, Shaanxi, China; ^3^ Institute of Biomedical Engineering, School of Life Science and Technology, Xi’an Jiaotong University, Xi’an, Shaanxi, China

**Keywords:** mendelian randomization, diabetes, glycemic traits, delirium, genetic

## Abstract

This study aims to explore the genetic causal association between type 2 diabetes (T2D) and glycemic traits (fasting glucose [FG], fasting insulin [FI], and glycated hemoglobin [HbA1c]) on delirium using Mendelian randomization (MR). Genome-wide association studies (GWAS) summary data for T2D and glycemic traits were obtained from the IEU OpenGWAS database. GWAS summary data for delirium were obtained from the FinnGen Consortium. All the participants were of European ancestry. In addition, we used T2D, FG, FI, and HbA1c as exposures and delirium as outcomes. A random-effects variance-weighted model (IVW), MR Egger, weighted median, simple mode, and weighted mode were used to perform MR analysis. In addition, MR-IVW and MR-Egger analyses were used to detect heterogeneity in the MR results. Horizontal pleiotropy was detected using MR-Egger regression and MR pleiotropy residual sum and outliers (MR-PRESSO). MR-PRESSO was also used to assess outlier single nucleotide polymorphisms (SNPs). The “leave one out” analysis was used to investigate whether the MR analysis results were influenced by a single SNP and evaluate the robustness of the results. In this study, we conducted a two-sample MR analysis, and there was no evidence of a genetic causal association between T2D and glycemic traits (T2D, FG, FI, and HbA1c) on delirium (all *p* > 0.05). The MR-IVW and MR-Egger tests showed no heterogeneity in our MR results (all *p* values >0.05). In addition, The MR-Egger and MR-PRESSO tests showed no horizontal pleiotropy in our MR results (all *p* > 0.05). The MR-PRESSO results also showed that there were no outliers during the MR analysis. In addition, the “leave one out” test did not find that the SNPs included in the analysis could affect the stability of the MR results. Therefore, our study did not support the causal effects of T2D and glycemic traits (FG, FI, and HbA1c) on delirium risk.

## 1 Introduction

Delirium is a distressing acute encephalopathy characterized by the acute onset of deficits in attention, awareness, and cognition that fluctuate in severity over time (Wilson et al., 2020). Delirium is the most common surgical complication among older adults, with an incidence of 15–25% after major elective surgery and 50% after high-risk procedures such as hip-fracture repair and cardiac surgery (Gibb et al., 2020). Delirium is associated with prolonged length of hospital stay and costs, higher morbidity and mortality, cognitive decline, dementia, and poorer overall outcomes (Gleason et al., 2015; Marcantonio, 2017; Mattison, 2020). With the impact of the recent and ongoing coronavirus disease pandemic, these costs are likely to have increased many times over (Wilson et al., 2020).

Some leading mechanisms postulated to contribute to delirium include neurotransmitters, inflammation, physiological stressors, metabolic derangements, electrolyte disorders, and genetic factors (Inouye et al., 2014; Oldham and Holloway, 2020). The lack of effective treatments calls for the identification of modifiable risk factors and strategies for prevention. In cohort studies, type 2 diabetes (T2D) shows associations with a higher risk for delirium independently of other risk factors (Milisen et al., 2020; Haynes et al., 2021; Liu et al., 2022), but still conflicting findings exist (Bramley et al., 2021). The differences in the study population, methodology, and surgery type may explain these different results. In addition, abnormal glycemic traits, including fasting glucose (FG), fasting insulin (FI), and hemoglobin A1c (HbA1c) levels, have been reported to be associated with delirium (Lin et al., 2021; Song et al., 2022). However, these studies were subject to various methodological limitations, such as a high risk of selection bias, unclear outcome definitions, or retrospective data collection.

No conclusion has yet been reached regarding the relationship between T2D, glycemic traits, and delirium. Mendelian randomization (MR) may help clarify these associations. The MR approach uses genetic information as an instrumental variable to address some of the limitations of observational studies and to estimate causality so that the results are generally independent of environmental confounders and less subject to reverse causation (Holmes et al., 2017; O’Donnell and Sabatine, 2018). In this study, we used large-scale genome-wide association studies (GWAS) data and performed a two-sample MR analysis to investigate the causal effect of T2D and related glycemic traits (FG, FI, and HbA1c) on delirium.

## 2 Materials and methods

### 2.1 Study design and data sources

GWAS summary data for T2D and glycemic traits were obtained from the IEU OpenGWAS database. The data of T2D included 12,931 patients and 57,196 controls, with a total of 14,277,791 single nucleotide polymorphisms (SNPs). FG data included 200,622 samples and 31,008,728 SNPs. FI data included 151,013 samples and 29,664,438 SNPs. The data for HbA1c levels included 146,806 samples and 30,649,064 SNPs. All participants were of European ancestry, and informed consent was obtained. Each participating cohort underwent study-level quality control (QC), imputation, and association analyses following a shared analysis plan.

Cohorts were genotyped using commercially available genome-wide arrays or the Illumina CardioMetabochip (Metabochip) array. Before imputation, each cohort underwent stringent sample and variant QC to ensure that only high-quality variants were retained in the genotype scaffold for imputation. Sample QC checks included removing samples with a low call rate of less than 95%, extreme heterozygosity, sex mismatch with X-chromosome variants, duplicates, first- or second-degree relatives (unless by design), or ancestry outliers. More details on the data can be found in the published study (Chen et al., 2021). GWAS summary data for delirium were obtained from the FinnGen Consortium. We used publicly available data from 1,269 patients with delirium and 209,487 controls of Finnish ancestry. A total of 16,380,452 SNPs were identified. All cases were defined using the code M13 in the International Classification of Diseases, 10th Revision (ICD-10). Detailed information on participants, genotyping, imputation, and QC can be found on the FinnGen website (http://finngen.gitbook.io/documentation/).

### 2.2 Instrumental variable selection

MR analysis of exposure and outcome was performed using strictly censored instrumental variables (IVs). We obtained SNPs that were strongly associated (*p* < 5 × 10^−8^, F > 10) with four exposures (T2D, FG, FI, and HbA1c levels). Because strong linkage disequilibrium (LD) among the selected SNPs may lead to biased results, the clumping process (*r* ^2^ < 0.001, clumping distance = 10,000 kb) was carried out to eliminate the LD between the included IVs (Chen et al., 2022). Furthermore, palindromic SNPs with intermediate allele frequencies were excluded to guarantee that the impact of SNPs on exposure corresponded to the same allele as the effect on outcome (Cao et al., 2022). In addition, we applied the PhenoScanner database (http://www.phenoscanner.medschl.cam.ac.uk/phenoscanner) to assess whether the selected SNPs were associated with other traits at the genome-wide significance levels (Shu et al., 2022). When SNPs were not available from GWAS results, proxy SNPs were identified using the online platform LDlink (https://ldlink.nci.nih.gov/).

### 2.3 Statistical analysis

The “TwoSampleMR” package of the R software (version 4.1.2) was used to perform two-sample MR analysis of exposure and outcome. We used a random-effects variance-weighted model (IVW), MR-Egger, weighted median, simple mode, and weighted mode to perform MR analysis (Cao et al., 2022). With random-effects IVW as the main method and weighted median, simple mode, and weighted mode as supplementary methods. We used the I^2^ index and Cochran’s Q statistic for MR-IVW analyses and Rucker’s Q statistic for MR-Egger analyses to detect heterogeneity of the effects of SNPs related to T2D, FG, FI, and HbA1c on delirium, and *p* > 0.05, indicating no heterogeneity (Hemani et al., 2018). We used the MR-Egger method to test for horizontal pleiotropy, and *p* > 0.05, indicating no horizontal pleiotropy (Shu et al., 2022). Since MR-Egger may show lower accuracy in some cases, the MR pleiotropy residual sum and outlier (MR-PRESSO) method was also used to assess outlier SNPs and potential horizontal pleiotropy. We also performed a ‘leave one out’ analysis to investigate whether the causal relationship between exposure and outcome was influenced by a single SNP (Lee, 2019).

## 3 Results

### 3.1 Instrumental variable selection

After a series of quality controls, we obtained 14 SNPs as IVs for MR analysis of T2D and delirium (Supplementary Table S1). We obtained 59 SNPs as IVs for MR analysis of FG and delirium, among which there were 3 palindrome SNPs (Supplementary Table S2). We obtained 20 SNPs as IVs for the MR analysis of FI and delirium (Supplementary Table S3). We obtained 21 SNPs as IVs for MR analysis of HbA1c levels and delirium (Supplementary Table S4). Among the IVs obtained, none of the SNPs were proxied.

### 3.2 Results of mendelian randomization analysis

**FIGURE 1:**
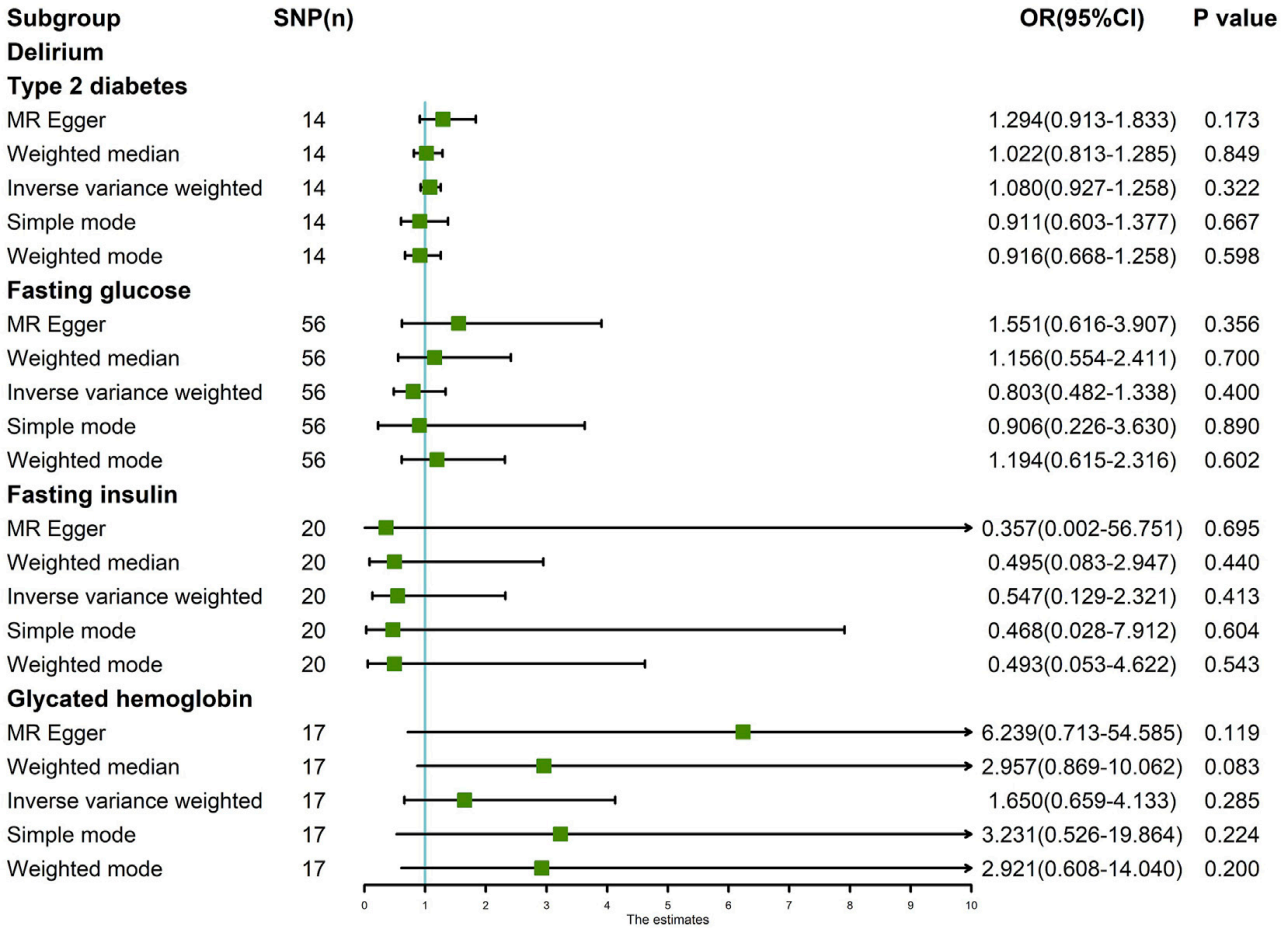
MR analysis results of the four exposures (T2D, FG, FI, and HbA1c) and outcome (delirium).

**FIGURE 2:**
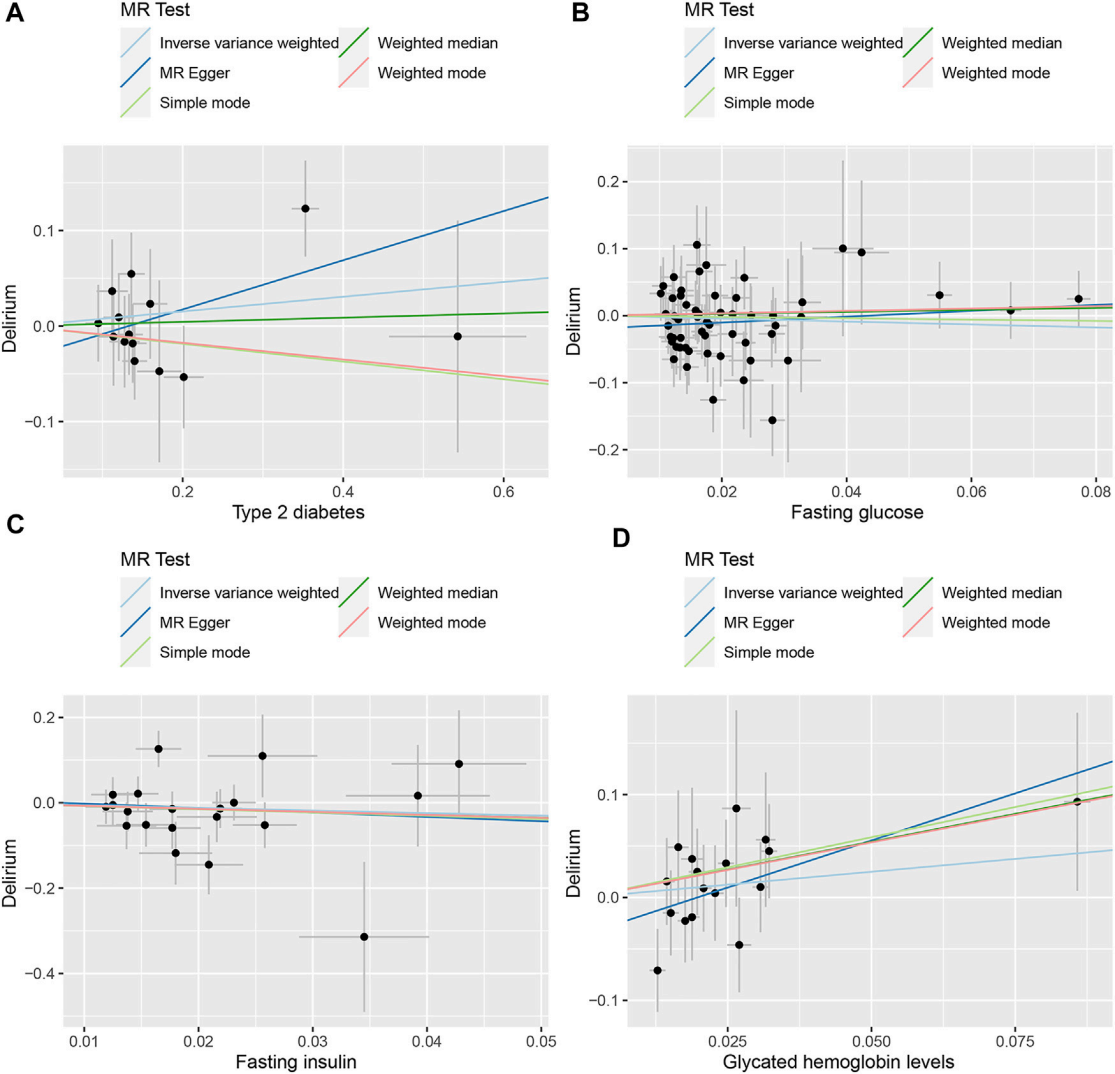
Scatter plot of the MR results between exposures and outcome. **(A)** T2D and delirium. **(B)** FG and delirium. **(C)** FI and delirium. **(D)** HbA1c and delirium.

The random-effects IVW results suggest that T2D (*p* = 0.322, odds ratio [OR] (95% confidence interval [CI]) = 1.080 [0.927–1.258]), FG (*p* = 0.400, OR [95% CI] = 0.803 [0.482–1.338]), FI (*p* = 0.413, OR [95% CI] = 0.547 [0.129–2.321]), and HbA1c (*p* = 0.427, OR [95% CI] = 1.428 [0.592–3.445]) have no genetic causal relationship with delirium. In addition, MR-Egger, weighted median, simple mode, and weighted mode analyses also showed that T2D, FG, FI, and HbA1c had no genetic causal relationship with delirium (Figures 1, 2).

**FIGURE 3:**
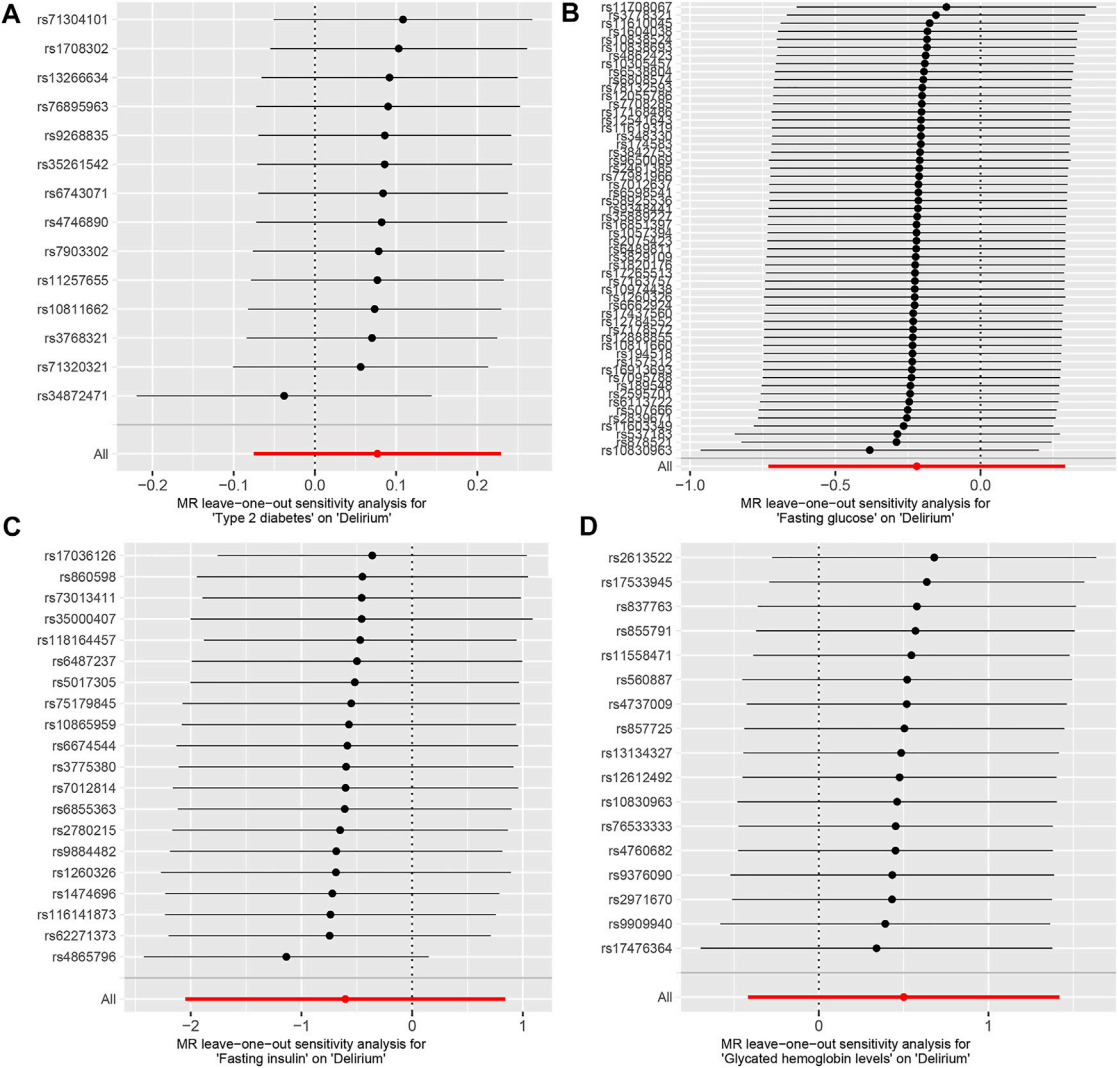
Leave one out analysis of the MR results between exposures and outcome. **(A)** T2D and delirium. **(B)** FG and delirium. **(C)** FI and delirium. **(D)** HbA1c and delirium.

The IVW test showed no heterogeneity in the MR analysis results for T2D (*p* = 0.717), FG (*p* = 0.634), FI (*p* = 0.144), and HbA1c (*p* = 0.889) with delirium. Likewise, the MR-Egger test showed no heterogeneity in the MR analysis results for T2D (*p* = 0.750), FG (*p* = 0.702), FI (*p* = 0.112), and HbA1c (*p* = 0.932) with delirium. The MR-Egger test showed no horizontal pleiotropy in the MR analysis results for T2D (*p* = 0.281), FG (*p* = 0.099), FI (*p* = 0.865), and HbA1c (*p* = 0.205) with delirium (Table 1). The results of MR-PRESSO showed no horizontal pleiotropy in the MR analysis of T2D (*p* = 0.552), FG (*p* = 0.665), FI (*p* = 0.149), and HbA1c (*p* = 0.902) with delirium. The MR-PRESSO results showed no outliers during the MR analysis (Table 1). In addition, the “leave-one-out” analysis showed that the results of our MR analysis were not affected by a single SNP (Figure 3).

**TABLE 1:** Sensitivity analysis of the MR analysis results of exposures and outcomes.

Exposure	Outcome	Heterogeneity test		Pleiotropy test	MR-PRESSO	
		Cochran’s Q test (P value)	Rucker’s Q test (P value)	Egger intercept (P value)	Distortion test	Global test
		IVW	MR-egger	MR-egger	Outliers	P Value
Type 2 diabetes	Delirium	0.717	0.750	0.281	NA	0.552
Fasting glucose	Delirium	0.634	0.702	0.099	NA	0.665
Fasting insulin	Delirium	0.144	0.112	0.865	NA	0.149
Glycated hemoglobin	Delirium	0.889	0.932	0.205	NA	0.902

## 4 Discussion

By leveraging large-scale GWAS data in MR analysis, we investigated the causal associations between T2D, glycemic traits, and delirium. There was no evidence of positive or negative genetic causality between T2DM, FG, FI, and HbA1c with delirium. The *F* values of IVs indicate that the variables satisfy the strong correlation assumption of MR analysis, and the instrument bias is weak; therefore, estimates of causal effects are not materially affected. In addition, we used MR-IVW and MR-Egger analyses to detect heterogeneity, and MR-Egger regression and MR-PRESSO were used to detect horizontal pleiotropy. MR-PRESSO was also used to assess outlier SNPs. The results showed no heterogeneity, horizontal pleiotropy, or outliers. Furthermore, the “leave one out” analysis showed that our MR analysis results were not affected by a single SNP, which indicated the reliability of the MR results.

Although the exact mechanism of delirium is not known, it is generally thought to involve abnormal nerve transmission and neuroinflammation (Inouye et al., 2014; Oldham and Holloway, 2020). It has been suggested that cumulative neuroinflammation and neurodegeneration resulting from oxidative stress caused by chronic hyperglycemia in patients with T2D might result in progressive structural abnormalities in the brain that can lead to delirium (Rom et al., 2019). The current observational studies concerning the association between T2D and delirium show conflicting results (Bramley et al., 2021). In addition to differences in population, methods, and type of surgery, these prior studies may have been subject to other confounding factors, did not define diabetes and did not report information on glycemic control. Considering these residual confounders, the strength of the association may be heterogeneous, and the causal association between T2D and delirium has not yet been determined. Hence, conducting a more in-depth study on the correlation between T2D and delirium at the genetic level is necessary.

No previous studies have investigated the association between T2D and delirium using large-scale GWAS data. Recent studies (Garfield et al., 2021; Ware et al., 2021) used MR approaches to estimate the effects of T2D on cognitive outcomes. Of note, a recent analysis using a cumulative genetic risk score for T2D as a valid instrument showed no non-causal association between a history of T2D and cognitive impairment or non-dementia in European ancestry. In addition, using IVW, the genetic liability for T2D was not associated with reaction time or visual memory. In our study, we performed MR analysis with random-effects IVW as the main method and weighted median, simple mode, and weighted mode as supplementary methods, and the results suggest that T2D has no genetic causal relationship with delirium. However, other factors may also play mediating roles. Important factors associated with T2D, such as low vitamin D levels, prior cognitive status, and cardiovascular complications, contribute to the development of delirium (Mattison, 2020; Wilson et al., 2020).

We also considered three glycemic traits (FG, FI, and HbA1c) closely related to T2D and conducted multivariable analyses to avoid bias of confounders caused by these traits. Several observational studies (van Keulen et al., 2018; Windmann et al., 2019; Song et al., 2022) have consistently found that perioperative acute hyperglycemia, independent of diabetes, is associated with delirium. Van Keulen et al. (van Keulen et al., 2018) was the first to explore the association between diabetes, glucose dysregulation, and their interplay in relation to delirium. They reported that glucose dysregulation was associated with the transition to intensive care unit (ICU) delirium in non-diabetic patients, and diabetes was not associated with an increased risk of ICU delirium. Similarly, Windmann et al. (Windmann et al., 2019), found that intraoperative hyperglycemia was associated with postoperative delirium independent of age, sex, diabetes, American Society of Anesthesiologists status, duration, and type of surgery; in particular, hyperglycemic non-diabetic patients might be at high risk for postoperative delirium. Furthermore, high preoperative HbA1c levels and poor glycemic control have been reported to increase the risk of postoperative delirium following cardiovascular surgery (Kotfis et al., 2019; Lin et al., 2021).

In clinical practice, hyperglycemia is common in hospitalized patients, and stress hyperglycemia is thought to be caused by inflammation and neurohormonal disturbances that occur during an acute illness. In fact, hyperglycemia not only results from poor control of chronic diabetes, but it can also be due to acute stress. Higher glycemic levels may reflect a more severe inflammatory state and neuroendocrine response, both of which are associated with the development of delirium (van Niekerk et al., 2019; Zhao et al., 2021).

Several studies (Arcambal et al., 2019; Rom et al., 2019; Watt et al., 2020) have reported that abnormally elevated glucose concentrations can promote the release of proinflammatory cytokines, disrupt the blood-brain barrier, and induce neuroinflammation, which may eventually lead to neural network disturbances that can induce delirium. However, it is unclear whether hyperglycemia is to blame or whether vascular risk factors (e.g., hypertension, dyslipidemia, and inflammation) mediate the link between diabetes and poorer brain function. We did not find evidence of a causal relationship between FG, FI, or HbA1c levels and the risk of delirium. Stress hyperglycemia usually results from inflammation and neuroendocrine disruption during acute illness; thus, it is likely that abnormal glycemic traits alone do not explain the increased risk of delirium in patients with T2D and could be a marker of vulnerability with diminished reserve capacity.

This study has several strengths. First, to the best of our knowledge, our study is the first to investigate the causal association between T2D and the related glycemic traits with delirium by leveraging large-scale GWAS. The two-sample MR method can overcome the limitations of some observational studies, such as reverse causality, confounding factors, and various biases. Second, to evaluate the robustness of the MR results, tests for heterogeneity and pleiotropy were conducted as additional means of sensitivity analysis.

However, some limitations of this study cannot be ignored. First, all the participants included in the GWAS were of European ancestry. Consequently, it remains to be determined whether our findings can be generalized to other populations and regions. Second, although we used the IVW and MR-Egger methods to detect and adjust for pleiotropy of genetic variants, there may still be confounding factors between exposure and outcome, such as level of education, personality, and nutrition that may have caued bias in our results. Third, only summary-level GWAS data were available, and the associated effects of sex, age, and specific exposure types on outcomes require further investigation.

## 5 Conclusion

In summary, the association between T2D and delirium is complex and dependent on multiple factors. However, given that the instrumental variable analysis findings are less likely to be biased than that of the observational estimates, our two-sample MR analysis did not suggest significant causal effects of T2D risk, FG, FI, and HbA1c on delirium.

## Data Availability

The datasets presented in this study can be found in online repositories. The names of the repository/repositories and accession number(s) can be found in the article/Supplementary Material.

## Abbreviations

FG, fasting glucose; FI, fasting insulin; GWAS, genome-wide association studies; HbA1c, glycated hemoglobin; ICU, intensive care unit; IVs, instrumental variables; IVW, random-effects variance-weighted model; LD, linkage disequilibrium; MR-PRESSO, MR pleiotropy residual sum and outliers; MR, Mendelian randomization; QC, quality control; SNPs, single nucleotide polymorphisms; T2D, type 2 diabetes.
